# Complete Genome Sequence of *Aeromonas* Phage ZPAH7 with Halo Zones, Isolated in China

**DOI:** 10.1128/MRA.01678-18

**Published:** 2019-03-07

**Authors:** Mohammad Sharifull Islam, Assaf Raz, Yang Liu, Khalid Ragaei Abdraboh Elbassiony, Xingxing Dong, Peng Zhou, Yang Zhou, Jinquan Li

**Affiliations:** a Key Laboratory of Environment Correlative Dietology, College of Food Science and Technology, Huazhong Agricultural University, Wuhan, Hubei, People’s Republic of China; b Laboratory of Bacterial Pathogenesis and Immunology, The Rockefeller University, New York, New York, USA; c College of Fisheries, State Key Laboratory of Agricultural Microbiology, Huazhong Agricultural University, Wuhan, Hubei, People’s Republic of China; d Plant Research Department, Nuclear Research Center, Atomic Energy Authority, Cairo, Egypt; Loyola University Chicago

## Abstract

Phage ZPAH7, isolated from a sediment sample from a fish farm, is a novel lytic phage belonging to the *Podoviridae* family. It produces large plaques (3.5 ± 0.2 mm) with halo zones (10.5 ± 0.5 mm), suggesting it has the ability to depolymerize exopolysaccharides and biofilms. It has a 30,791-bp genome encoding 29 putative coding sequences.

## Abstract (precis)

Phage ZPAH7, isolated from a sediment sample from a fish farm, is a novel lytic phage belonging to the *Podoviridae* family. It produces large plaques (3.5 ± 0.2 mm) with halo zones (10.5 ± 0.5 mm), suggesting it has the ability to depolymerize exopolysaccharides and biofilms.

## ANNOUNCEMENT

Aeromonas hydrophila is one of the key causative agents for fish mortality, resulting in substantial financial loss. No effective method has been proposed for the control of A. hydrophila infection in aquaculture, except for the application of additional antibiotics (1, 2). However, multiple-drug-resistant A. hydrophila was found to contribute to the high rate of mortality in the fish industry (3, 4). This study aimed to isolate phage to control A. hydrophila infection.

Phage ZPAH7 was isolated from a sediment sample from a fish farm in Wuhan, China. Ten microliters of a 0.22-µm-filtered sample was mixed with 40 ml 2-YT broth medium and 10 ml exponential-growth phase A. hydrophila ZYAH75. After 24 h of incubation at 25°C, the culture was centrifuged (8000 × *g* for 15 min) and filtered again using 0.22-µm filters (Millipore, Ireland). Then, phage activity in the supernatant was detected with the spot assay. This phage was a novel lytic phage with large plaques (3.5 ± 0.2 mm) compared with those of other phages (0.38 to 1.6 mm) (1, 5) and with halo formation (a lightening zone of 10.5 ± 0.5 mm). Halo formation has been linked to exopolysaccharide depolymerization and biofilm degradation (6, 7). The DNA was extracted, and the library was prepared using an Illumina TruSeq DNA nano library prep kit, according to the manufacturer’s instructions (8). The genome was sequenced using the Illumina HiSeq 4000 platform with a paired-end read length of 2 × 150 bp, producing 6,683,226 reads at >200× coverage. Quake (9) and BWA (10) were used in preassembly and postassembly sequence correction, respectively, and the genome was assembled with MicrobeTracker plus (v0.9.1) software.

The genomic length of phage ZPAH7 was 30,791 bp, with a G+C content of 56.8%. The genome results indicated that phage ZPAH7 belongs to the *Podoviridae* family, in the order of Caudovirales. BLAST analysis indicated that the genome sequence of phage ZPAH7 was related to that of *Aeromonas* phage phiAS7 (GenBank accession number JN651747), with a coverage of 91% (similarity, 81%). No data are available on the size of the plaques of phiAS7 and whether halo formation was observed, and thus, it is unknown whether this phage has the ability to degrade exopolysaccharides. The gene encoding the tail fiber protein of ZPAH7 was considerably different from that of phiAS7, sharing only 18% amino acid identity, as determined by BLASTP comparative analysis. This divergence in sequence may indicate that the host range of the two phages is different. The annotation results from the Rapid Annotations using Subsystem Technology (RAST) server (11) predicted 29 putative open reading frames (ORFs). Among the 29 ORFs, 20 ORFs were predicted to encode proteins with known functions. The products of the 12 ORFs belonged to the structure and packaging module (tail fiber protein, tail tubular protein, major capsid protein, scaffolding protein, head portal protein, and host specificity protein A), the products of the 6 ORFs belonged to the replication/transcription module (DNA maturase, DNA-dependent RNA polymerase, DNA endonuclease, DNA exonuclease, and DNA polymerase), and the products of the 2 ORFs belonged to the host lysis module. No virulence factors were identified by the Virulence Factor Database (see http://www.mgc.ac.cn/VFs/main.htm), and no antibiotic resistance genes were found using the Antibiotic Resistance Gene Database (12). The above evidence suggests that phage ZPAH7 could be used as a potential controlling agent against A. hydrophila.

### Data availability.

The raw sequences are available in the NCBI SRA database under BioProject number PRJNA517350. The complete genome sequence has been deposited in GenBank under the accession number MH992513.
